# Multiple Posterior Circulation Infarctions in a Patient With Complex Aortic Atheroma and Left Vertebral Artery Stenosis: A Case Report

**DOI:** 10.7759/cureus.115211

**Published:** 2026-08-26

**Authors:** Mohamed El Yamani, Karima Chokri, Lamiae Iben Brahim, Abdoulaye Camara, Benaissa Agraou

**Affiliations:** 1 Department of Cardiology, Ibn Sina University Hospital, Rabat, MAR; 2 Department of Cardiology, Centre Hospitalier de Maubeuge, Maubeuge, FRA

**Keywords:** aortic atheromatosis, cerebral embolism, complex aortic plaque, ischemic stroke, posterior circulation, transesophageal echocardiography

## Abstract

Complex aortic atheroma is a recognized potential source of cerebral embolism, particularly when plaques are thick, protruding, irregular, ulcerated, or mobile. However, establishing a direct causal relationship between aortic atheroma and cerebral infarction remains challenging when competing arterial mechanisms coexist. We describe a 65-year-old man with hypertension, dyslipidemia, and active tobacco use who presented with headache and an acute confusional state. Neurological examination revealed left-sided dysmetria and gait instability. Brain imaging demonstrated multiple ischemic lesions confined to the posterior circulation, involving the left cerebellum, left thalamus, and bilateral occipital regions. Computed tomography angiography revealed subocclusive stenosis of the left V4 vertebral artery, a right vertebral artery terminating as the posterior inferior cerebellar artery, and an irregular atheromatous plaque involving the distal aortic arch and isthmus. Transesophageal echocardiography demonstrated diffuse aortic atheromatosis, including a protruding distal aortic arch plaque measuring 26.03 × 12.79 mm and a descending aortic plaque with a maximum thickness of 12.35 mm. Transthoracic echocardiography, telemetry, and 72-hour Holter monitoring identified no cardiac embolic source or arrhythmia. Given the exclusively posterior infarct distribution and vertebral anatomy, symptomatic left V4 atherosclerotic stenosis, through artery-to-artery embolism, distal hypoperfusion, or both, was considered the more likely mechanism. The patient received dual antiplatelet therapy followed by long-term aspirin, high-intensity statin therapy, and intensive vascular risk-factor management, with favorable neurological improvement. The aortic atheroma may have been contributory, incidental, or a marker of systemic atherosclerosis rather than the direct embolic source. As definitive causal attribution was not possible, the stroke was classified as being of undetermined etiology under the TOAST framework.

## Introduction

Aortic atheromatosis is a potential source of systemic and cerebral embolism, particularly when plaques are thick, irregular, ulcerated, or contain a mobile component [1-3]. Lipid-rich plaques are considered more unstable and more likely to promote thrombus formation than heavily calcified lesions. An aortic plaque is generally classified as complex and associated with a higher embolic risk when it is more than 4 mm thick or exhibits ulceration or a mobile component [1-3].

Nevertheless, establishing a direct causal relationship between aortic arch atheroma and cerebral infarction may be difficult, particularly when another plausible arterial source coexists [2,3]. When infarctions are confined to the posterior circulation, careful assessment of the vertebral and basilar arteries is essential to identify a potentially competing vertebrobasilar mechanism.

Transesophageal echocardiography plays an important role in this diagnostic workup [1,4]. Owing to its high spatial resolution, it allows detailed assessment of the aortic arch and descending thoracic aorta, including plaque location, thickness, morphology, and potential mobility [1,4]. Three-dimensional transesophageal echocardiography may further improve the detection of complex plaques, particularly ulcerated lesions [1,4].

We report the case of a patient with multiple ischemic infarctions confined to the posterior circulation. The etiological workup identified both subocclusive intracranial left vertebral artery stenosis and diffuse complex atheromatosis of the distal aortic arch and descending thoracic aorta. Through this case and a focused narrative discussion, we examine the difficulty of causal attribution when two plausible atherosclerotic abnormalities coexist.

## Case presentation

A 65-year-old man with modifiable cardiovascular risk factors, including active chronic smoking and well-controlled hypertension treated with dual antihypertensive therapy, was admitted to the emergency department for headache and an acute confusional state. His medical history included COVID-19 infection three years earlier and an episode of vertigo associated with gait instability three months previously, during which brain magnetic resonance imaging showed no evidence of stroke.

At approximately 7:00 p.m., the patient reported headache and inappropriate speech during a telephone call with his son. He presented to the emergency department at approximately 2:00 a.m. with persistent headache and acute confusion; his admission NIHSS (National Institutes of Health Stroke Scale)** **score was 1. Intravenous thrombolysis was not administered because of the approximately seven-hour delay, and thrombectomy was not performed because no suitable target occlusion was identified.

Admission tests showed a normal blood count (hemoglobin, 14 g/dL), serum electrolytes, and liver function, with a C-reactive protein level of 1.3 mg/L. Glycated hemoglobin (HbA1c) was 5.3%. Total cholesterol was 2.10 g/L, HDL (high-density lipoprotein) cholesterol was 0.32 g/L, LDL (low-density lipoprotein) cholesterol was 1.28 g/L, and triglycerides were 2.51 g/L.

On clinical examination, his heart rate was 72 beats per minute with a regular rhythm, and his blood pressure was 136/78 mmHg. Cardiovascular examination revealed no cardiac murmur or additional heart sounds and no signs of left- or right-sided heart failure.

Neurological examination showed an alert patient with left-sided finger-to-nose dysmetria and gait instability associated with mild widening of the base of support. There was no motor or sensory deficit and no oculomotor palsy. The remainder of the clinical examination was unremarkable.

**Figure 1:**
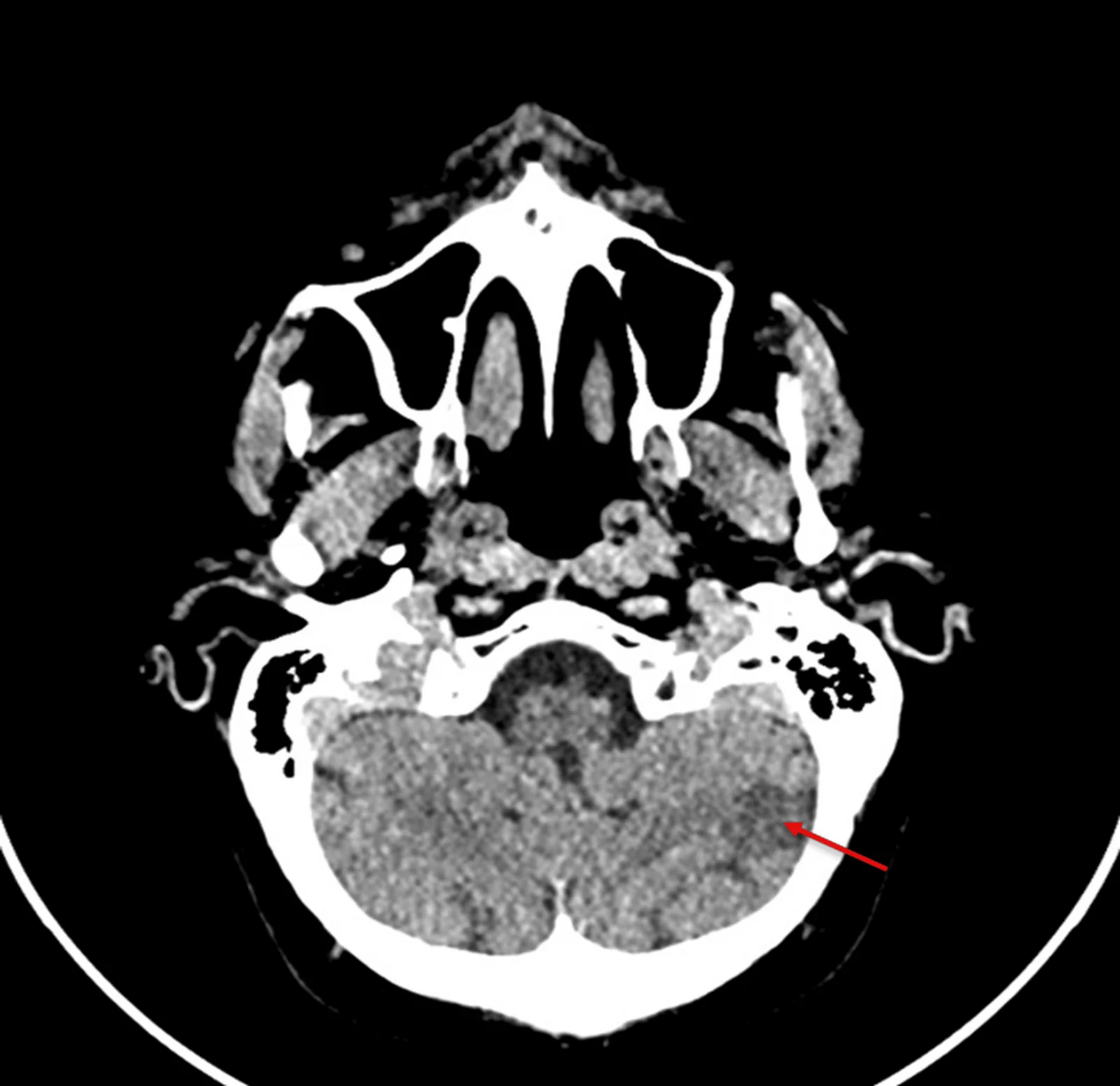
Non-contrast head computed tomography showing a left cerebellar hypodensity (red arrow)

Electrocardiography showed normal sinus rhythm. Inpatient telemetry detected no atrial fibrillation or other sustained arrhythmia. A 72-hour Holter monitoring demonstrated only occasional isolated supraventricular and ventricular premature beats, without atrial fibrillation or conduction abnormalities. Prolonged rhythm monitoring beyond 72 hours was not performed; therefore, paroxysmal atrial fibrillation could not be definitively excluded. Brain computed tomography performed on admission showed a left cerebellar hypodensity, most likely of vascular origin (Figure 1).

**Figure 2:**
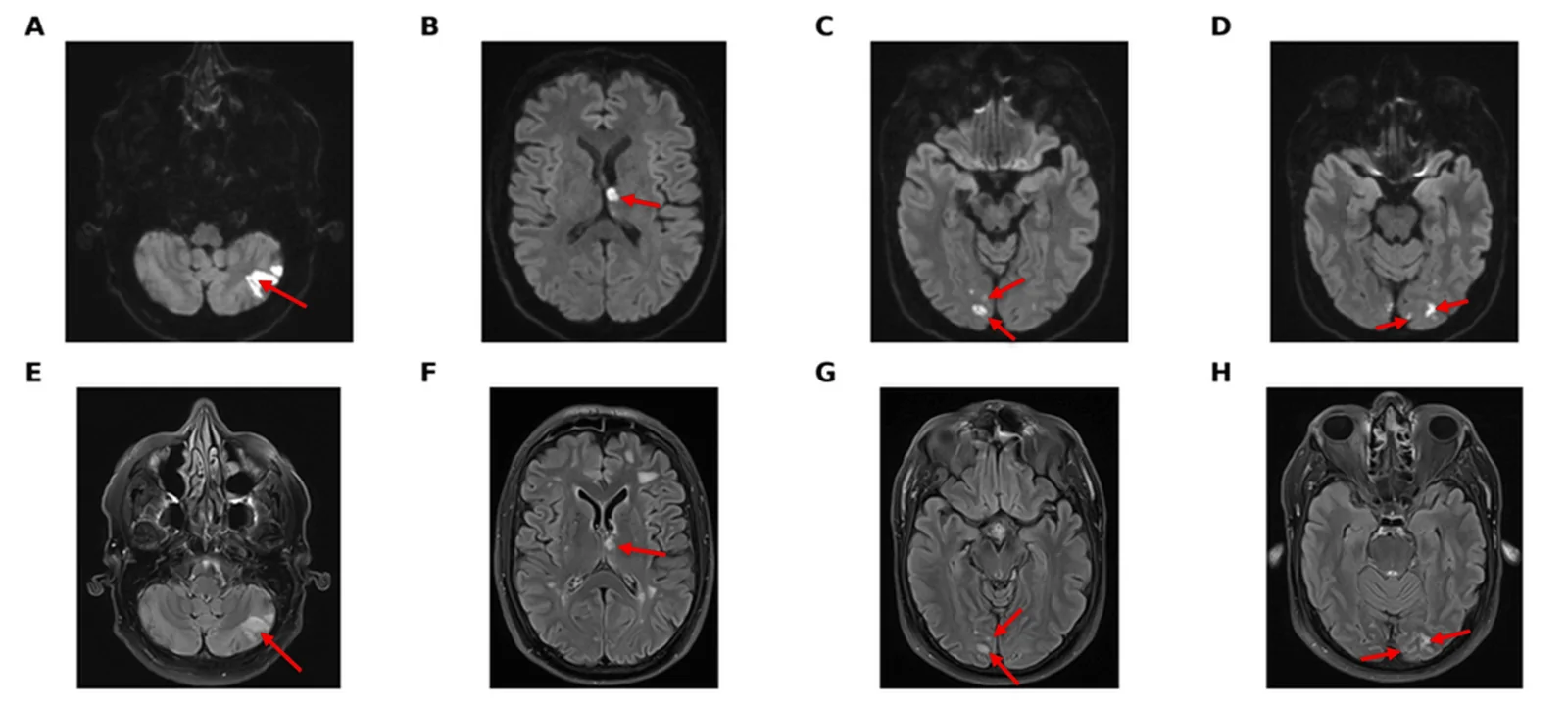
Brain magnetic resonance imaging demonstrating ischemic lesions confined to the posterior circulation. Diffusion-weighted images show lesions in the left cerebellum (A), left thalamus (B), right occipital lobe (C), and left occipital lobe (D). Corresponding FLAIR images show lesions in the left cerebellum (E), left thalamus (F), right occipital lobe (G), and left occipital lobe (H). The arrows indicate the ischemic lesions. DWI: Diffusion-weighted imaging; FLAIR: Fluid-attenuated inversion recovery.

Brain magnetic resonance imaging demonstrated hyperintense ischemic lesions on diffusion-weighted imaging involving the left cerebellum, left thalamus, and bilateral occipital regions, with corresponding abnormalities on fluid-attenuated inversion recovery imaging (Figure 2).

All identified lesions were confined to the posterior circulation. Duplex ultrasonography of the cervical vessels showed minimal atheromatous involvement of the carotid arteries, particularly on the left side, resulting in approximately 40% luminal narrowing without hemodynamic significance. Dampened flow was observed in the basilar artery and posterior cerebral arteries.

**Figure 3:**
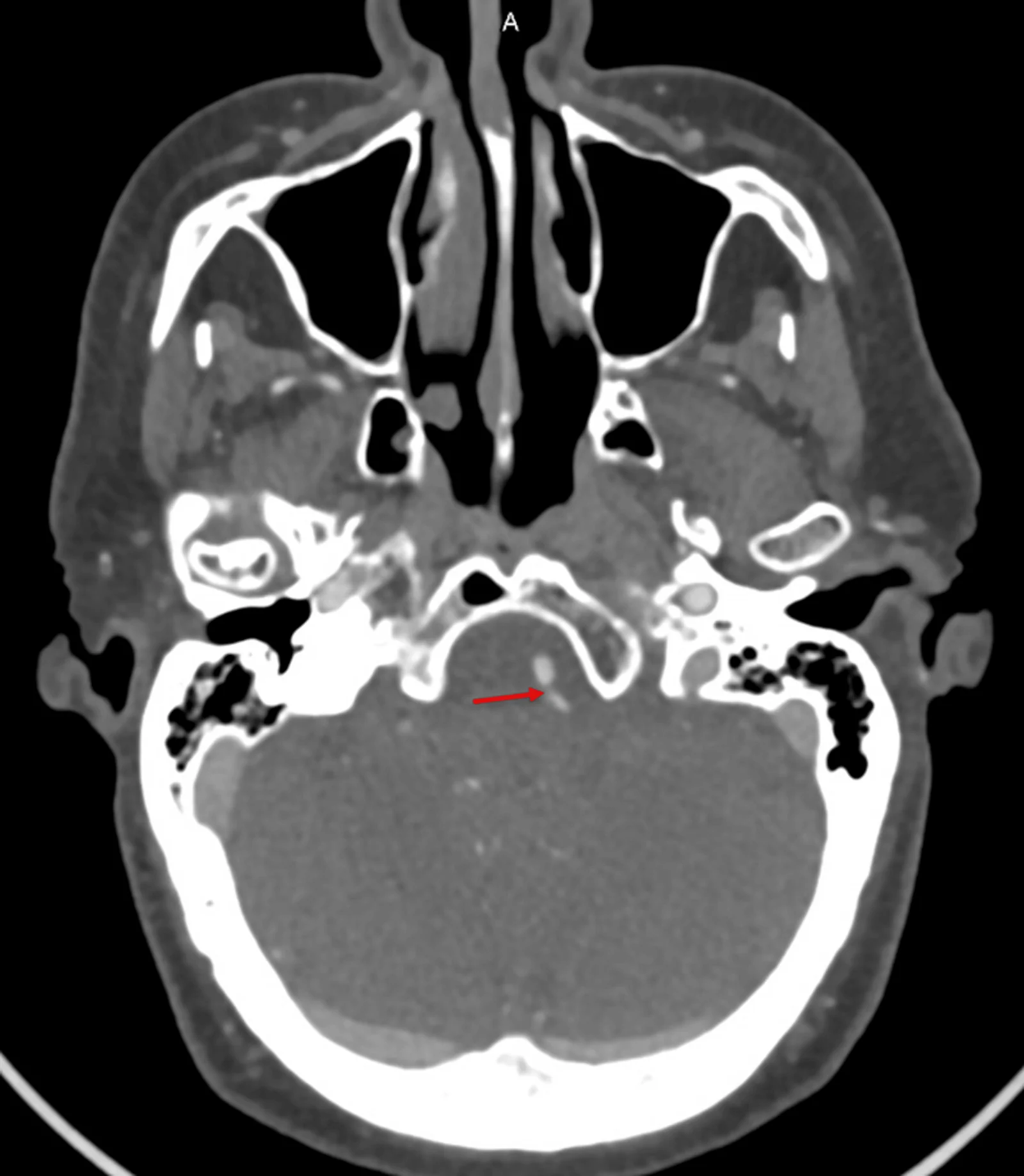
Computed tomography angiography of the supra-aortic and intracranial vessels showing subocclusive stenosis of the left V4 vertebral artery segment (arrow)

Computed tomography angiography of the supra-aortic and intracranial vessels revealed an established ischemic lesion in the vertebrobasilar territory, associated with subocclusive stenosis of the left V4 segment and a right vertebral artery terminating in the posterior inferior cerebellar artery (Figure 3).

**Figure 4:**
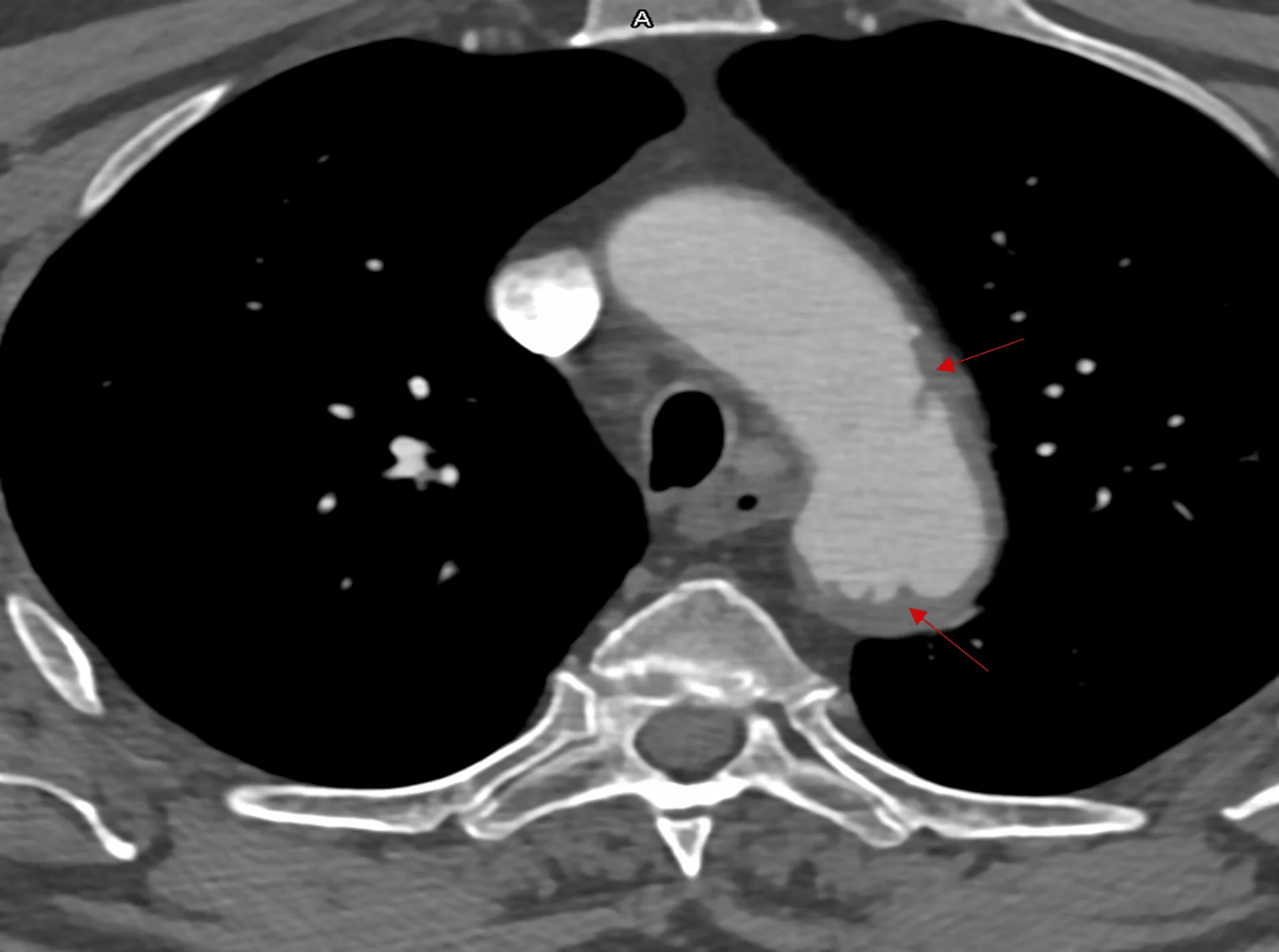
Computed tomography angiography showing an irregular atheromatous plaque involving the aortic arch and aortic isthmus (red arrows)

It also demonstrated an irregular atheromatous plaque involving the distal aortic arch and aortic isthmus, downstream from the origin of the left subclavian artery. Its exact distance from the left subclavian ostium was not retrospectively available. No aneurysmal dilatation or dissection of the thoracic aorta or major supra-aortic vessels was identified (Figure 4).

No dedicated flow study was performed to assess retrograde diastolic flow from the downstream aortic plaque toward the supra-aortic vessels; therefore, its causal relationship with the cerebral infarctions could not be established.

Despite active smoking, the patient had no history or clinical manifestations of peripheral arterial disease, including intermittent claudication, ischemic rest pain, peripheral ulceration, or prior peripheral revascularization. Peripheral pulses were palpable and symmetrical, with no clinical signs of limb ischemia.

**Figure 5:**
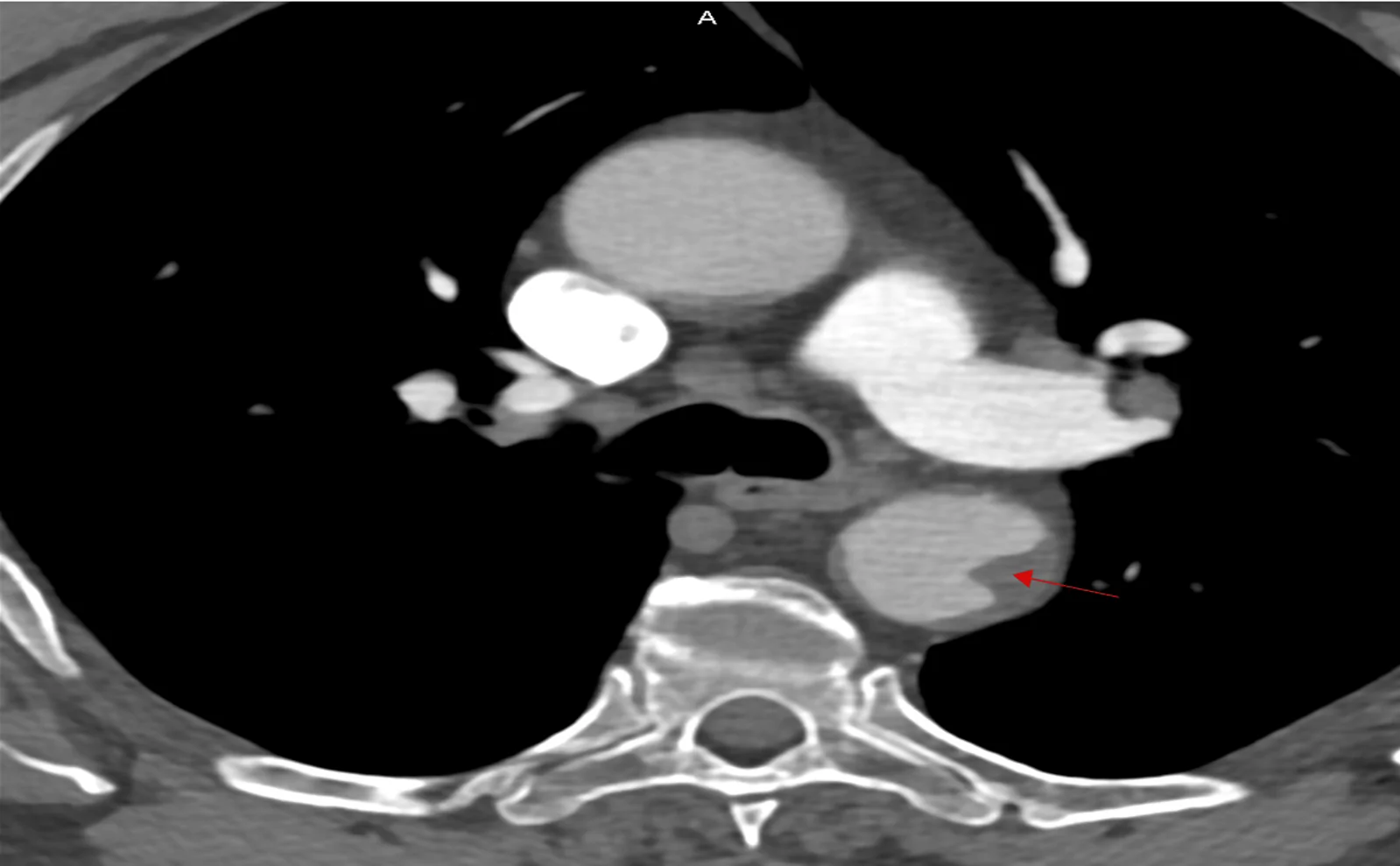
Computed tomography image showing an atheromatous plaque in the descending thoracic aorta (red arrow)

The patient had no history of angina or acute coronary syndrome. The electrocardiogram showed no ischemic changes, and cardiac troponin levels were within the normal range. Thoraco-abdominopelvic computed tomography demonstrated an atheromatous plaque in the descending thoracic aorta (Figure 5).

No abnormality was detected in the abdominal or pelvic regions. Transthoracic echocardiography demonstrated preserved left ventricular systolic function, with a left ventricular ejection fraction of 65%, normal left atrial size, no regional wall-motion abnormalities, no intracardiac thrombus, and no significant valvular abnormalities. The agitated saline contrast study was negative.

**Figure 6:**
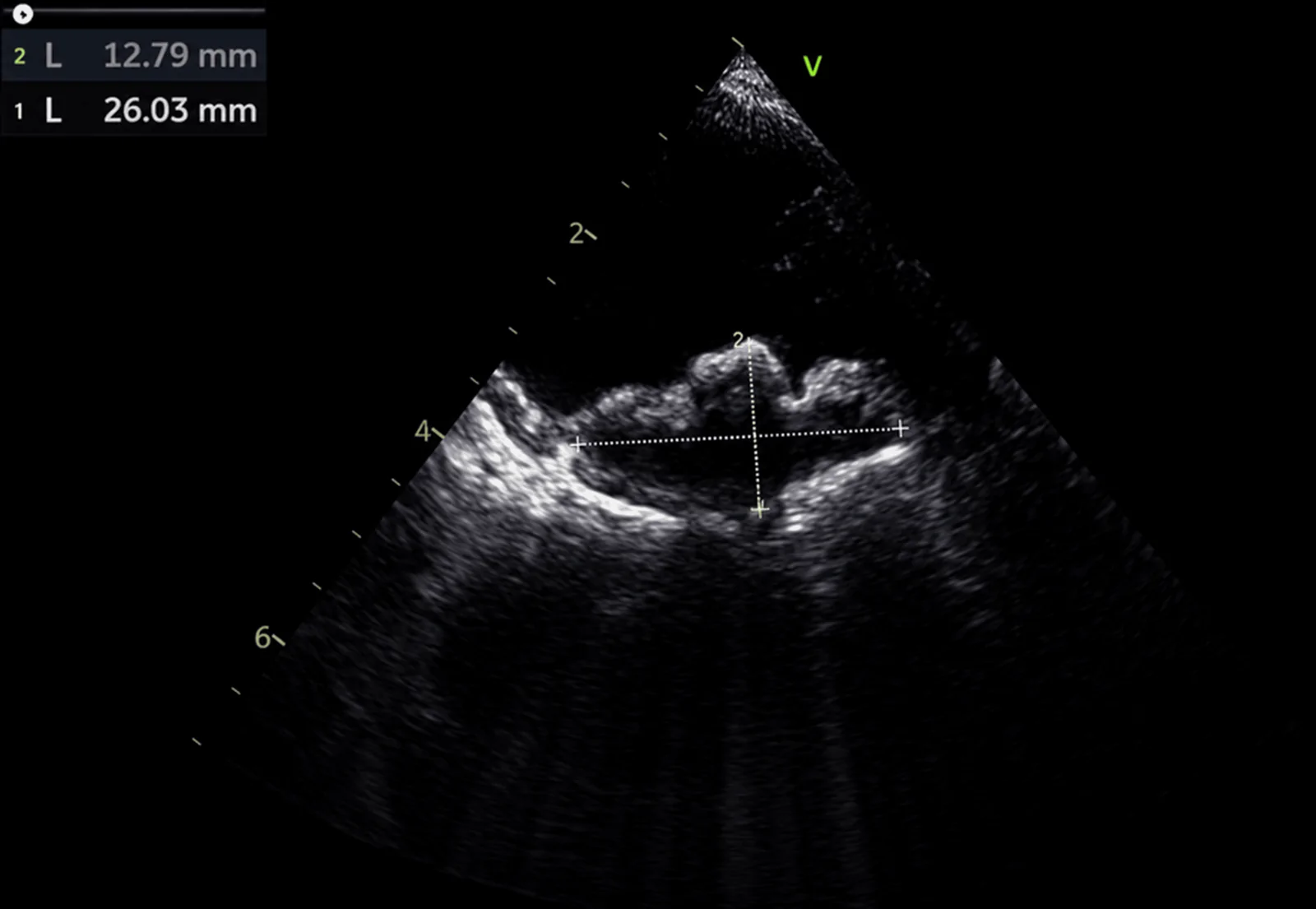
Transesophageal echocardiographic transverse view of the distal aortic arch showing a large, markedly irregular, protruding atheromatous plaque measuring 26.03 × 12.79 mm

**Figure 7:**
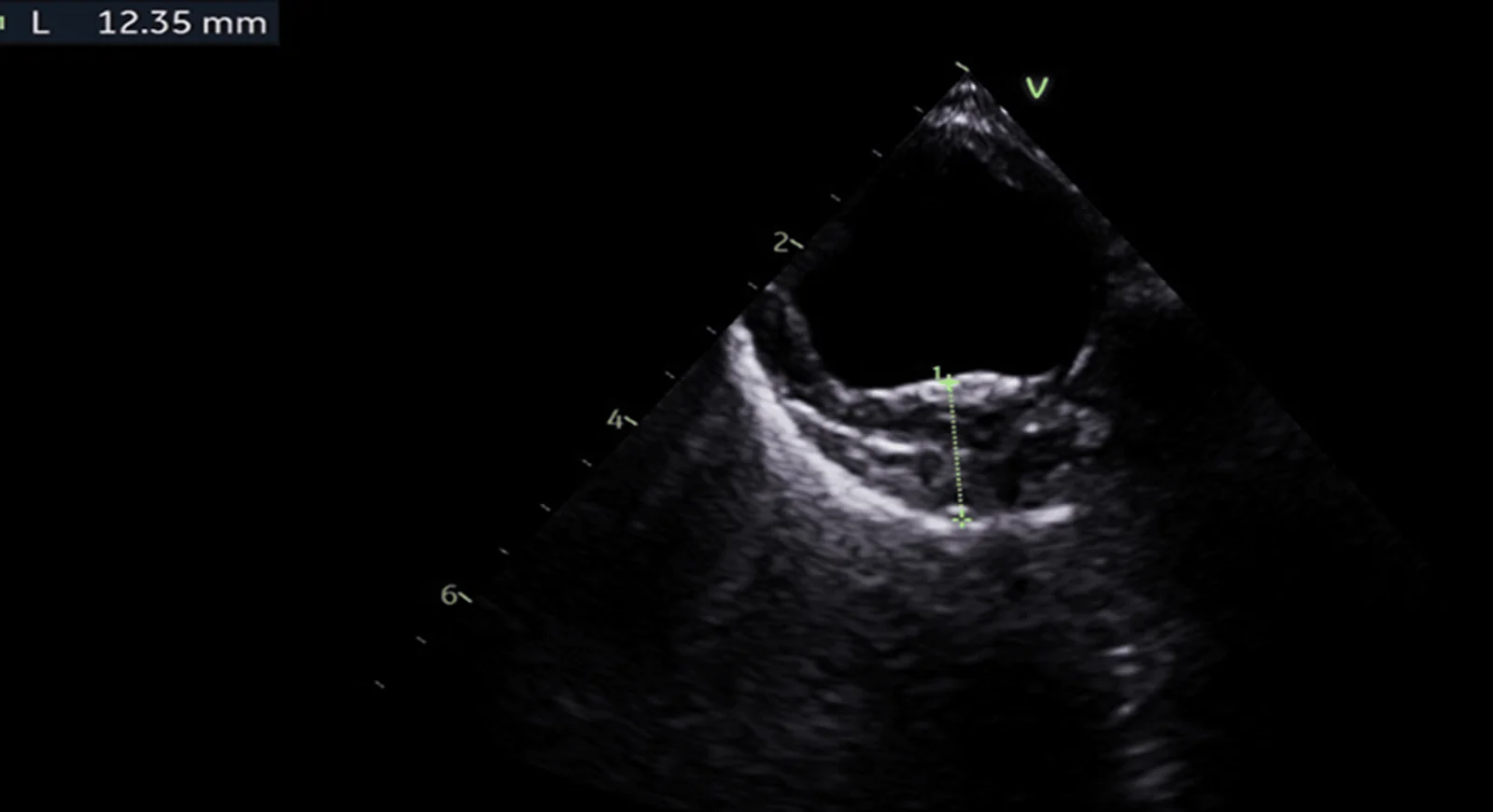
Transverse view of the descending thoracic aorta on transesophageal echocardiography, showing a large irregular mural atheromatous plaque with a maximum thickness of 12.35 mm

Transesophageal echocardiography showed no evidence of an interatrial shunt on color Doppler imaging or agitated saline contrast testing. The left atrial appendage was free of thrombus, and no spontaneous echo contrast was observed. It revealed diffuse aortic atheromatosis, with multiple irregular mural plaques involving the aortic arch and descending thoracic aorta. Two large protruding atheromatous plaques were identified, one in the aortic arch and the other in the descending thoracic aorta, without definite ulceration or a mobile component (Figures 6, 7).

Given the undetermined stroke mechanism and the coexisting subocclusive left V4 stenosis, dual antiplatelet therapy was initiated on admission with loading doses of aspirin 250 mg administered intravenously and clopidogrel 300 mg administered orally, followed by aspirin 75 mg once daily and clopidogrel 75 mg once daily. Dual antiplatelet therapy was continued for 15 days, including after discharge. Clopidogrel was subsequently discontinued, while aspirin 75 mg once daily was maintained as long-term antiplatelet therapy. No hemorrhagic transformation was identified on brain imaging. The detailed rationale for selecting a 15-day dual-antiplatelet regimen was not retrospectively available. As the left V4 stenosis was not quantitatively assessed using a validated intracranial method, it could not be formally classified as 70%-99% stenosis. Additional treatment included atorvastatin 80 mg once daily, candesartan 8 mg once daily, hydrochlorothiazide 12.5 mg once daily, pantoprazole 40 mg once daily, and nicotine replacement therapy with one 21 mg/24-hour transdermal patch daily. Therapeutic anticoagulation was not initiated because no atrial fibrillation, intracardiac thrombus, mobile aortic thrombus, venous thromboembolism, or other established indication for anticoagulation was identified. Home-based motor rehabilitation, complete smoking cessation, and regular physical activity consisting of 45 minutes of walking three times per week were also recommended.

After one week of hospitalization, the patient’s clinical course was favorable, with resolution of the confusional state and recovery of full orientation to time and place. However, amnesia for the acute episode persisted, particularly regarding the circumstances surrounding his admission. He was discharged with a modified Rankin Scale score of 2 and continuation of the prescribed treatment and home-based rehabilitation plan. No standardized post-discharge follow-up data were available (Table 1). Written informed consent was obtained from the patient for publication of this case report and the accompanying clinical images.

**Table 1:** CARE-style timeline of the clinical course CARE: CAse REports Guidelines; CTA: Computed tomography angiography; MRI: Magnetic resonance imaging; NIHSS: National Institutes of Health Stroke Scale; PICA: Posterior inferior cerebellar artery; TEE: Transesophageal echocardiography; TTE: Transthoracic echocardiography; mRS: Modified Rankin scale.

Time point	Main events
Three months before admission	Vertigo and gait instability; brain MRI showed no infarction.
7:00 p.m.	Onset of headache and inappropriate speech.
2:00 a.m.	Emergency admission approximately seven hours after onset; NIHSS score 1. No thrombolysis because of delayed presentation and no thrombectomy because no suitable target occlusion was identified.
During hospitalization	Multifocal posterior-circulation infarctions, subocclusive left V4 stenosis, and complex aortic plaques were identified. Cardiac investigations and 72-hour Holter monitoring detected no cardiac embolic source or atrial fibrillation. Dual antiplatelet therapy and atorvastatin were initiated.
Day 7	Confusion resolved, but amnesia persisted. Discharge mRS score was 2, with home-based rehabilitation.
After discharge	Dual antiplatelet therapy was continued for a total of 15 days, followed by long-term aspirin. No standardized follow-up data were available.

## Discussion

Thoracic aortic atheromatosis is recognized as a potential source of cerebral embolism, although establishing a causal relationship between stroke and an aortic plaque may be difficult because of the high prevalence of atherosclerosis in older patients and the possible coexistence of other embolic or arterial mechanisms. In our patient, however, all identified infarcts were confined to the posterior circulation. Combined with the subocclusive left V4 stenosis and the termination of the right vertebral artery as the posterior inferior cerebellar artery, this distribution makes symptomatic left vertebral artery disease more likely than an aortic source. Electrocardiography, telemetry, transthoracic echocardiography, and agitated saline contrast testing did not identify an obvious cardioembolic source.

Not all aortic plaques carry the same embolic risk. Lipid-rich, minimally calcified plaques infiltrated by inflammatory cells are considered more unstable than heavily calcified lesions [1]. Plaque rupture or ulceration may promote the formation of a mural thrombus that can subsequently fragment [1]. An aortic plaque is generally classified as complex when it is more than 4 mm thick or when it exhibits ulceration or a mobile thrombotic component [1].

In our patient, transesophageal echocardiography demonstrated diffuse atheromatosis of the aortic arch and descending thoracic aorta, with two major protruding plaques. The distal aortic arch plaque measured 26.03 × 12.79 mm and had a markedly irregular surface, whereas the descending aortic plaque reached a thickness of 12.35 mm. These dimensions greatly exceeded the 4-mm threshold commonly used to define a plaque at high embolic risk. Despite the absence of a formally documented mobile component, their thickness, intraluminal protrusion, and irregular morphology support their classification as complex aortic lesions. The large distal arch/isthmic plaque was located downstream from the origin of the left subclavian artery. Cerebral embolization from this plaque would therefore require retrograde diastolic flow connecting it to a supra-aortic vessel supplying the affected cerebral territory. However, its exact distance from the left subclavian ostium was not retrospectively available, and no dedicated assessment of retrograde aortic flow was performed. Consequently, retrograde embolization could not be demonstrated, and the plaque should be regarded as a potential but unproven embolic source and as a marker of extensive systemic atherosclerosis.

Complex aortic atheroma may cause cerebral infarction through embolization of thrombotic or atheromatous material [1-3]. Individual cases of cerebral embolization associated with large aortic arch thrombi have also been reported [5]; however, such reports do not establish a specific predilection for the posterior circulation. In the present case, the exclusively posterior distribution of the infarcts, together with the subocclusive left V4 stenosis and the termination of the right vertebral artery as the posterior inferior cerebellar artery, makes a vertebrobasilar atherosclerotic mechanism more plausible. An aortic embolic mechanism remains possible but cannot be definitively established, and the aortic atheroma may have been contributory, incidental, or a marker of systemic atherosclerosis.

Symptomatic intracranial left vertebral artery atherosclerotic stenosis was considered the most likely stroke mechanism. The right vertebral artery terminated as the posterior inferior cerebellar artery, leaving the left vertebral artery as the principal contributor to the basilar circulation. In this anatomical context, the subocclusive left V4 lesion could account for the cerebellar, thalamic, and bilateral occipital infarctions through artery-to-artery embolism, distal hypoperfusion, or a combination of both mechanisms. Intracranial atherosclerotic stenosis is an especially important cause of ischemic stroke in Asian populations, and its recognized risk factors include older age, hypertension, dyslipidemia, smoking, diabetes, and prior stroke [6]. Our patient had several of these risk factors, including age, hypertension, dyslipidemia, and active smoking. Nevertheless, the percentage of stenosis, residual lumen, lesion length, plaque morphology, distal flow, and collateral circulation were not quantified using a validated intracranial method. Under the TOAST framework, the stroke was classified as having an undetermined etiology because two plausible large-artery atherosclerotic abnormalities coexisted, although the left V4 lesion was considered more likely to be symptomatic.

Imaging plays a central role in the identification and characterization of aortic atheroma. Computed tomography angiography enables comprehensive assessment of the aorta and its branches and provides information on the calcified and noncalcified components of atherosclerotic plaques [1]. In our case, it revealed an irregular plaque involving the aortic arch and isthmus, as well as a second plaque in the descending thoracic aorta. Transesophageal echocardiography subsequently provided a more detailed characterization of plaque surface, thickness, protrusion, and extent. It is considered a reference imaging modality for the assessment of aortic plaques in patients presenting with a cerebrovascular event because of its spatial resolution and its ability to visualize most of the thoracic aorta [1,4]. Three-dimensional transesophageal echocardiography may further improve the detection of complex lesions, particularly ulcerations [1,4]. However, it remains semi-invasive and may provide suboptimal visualization of certain portions of the ascending aorta and aortic arch [1]. Computed tomography angiography and vascular magnetic resonance imaging therefore provide complementary information, with magnetic resonance imaging also allowing assessment of plaque morphology, the brain, and the cervicocephalic arteries [1,7]. In this case, the concordance between computed tomography angiography and transesophageal echocardiography supports the anatomical presence of aortic disease.

Beyond its potential embolic role, aortic atheromatosis should be regarded as a marker of systemic atherosclerotic disease. Complex plaques are associated with stroke, systemic embolism, major cardiovascular events, and increased mortality [1,3,8]. Their presence should prompt investigation for concomitant atherosclerotic involvement of the coronary, carotid, and peripheral arterial territories [1]. In our patient, the atherosclerotic burden appeared substantial despite only moderate carotid involvement without hemodynamic significance. His main predisposing factors were age, hypertension, dyslipidemia, and active smoking. In retrospect, the episode of vertigo associated with gait instability that occurred three months earlier may have represented a transient ischemic event involving the posterior circulation, although magnetic resonance imaging performed at that time showed no evidence of infarction. However, the available findings do not establish a relationship between that previous episode and the infarctions identified during the current admission.

Management is primarily based on intensive cardiovascular risk reduction, including smoking cessation, strict blood pressure control, lipid-lowering therapy, an appropriate diet, and physical activity compatible with the patient’s neurological status [1]. High-intensity statin therapy is justified in patients with ischemic stroke and aortic plaques measuring more than 4 mm, while antiplatelet therapy is frequently used in this setting [1]. However, the available evidence does not support routine therapeutic anticoagulation solely on the basis of an aortic plaque. In the absence of atrial fibrillation, a mobile aortic thrombus, a prosthetic heart valve, or another established indication for anticoagulation, secondary vascular prevention should therefore be based on antiplatelet therapy, intensive lipid-lowering treatment, smoking cessation, and comprehensive cardiovascular risk-factor control [1]. The choice and duration of antithrombotic therapy should also take into account the severity of any symptomatic intracranial stenosis and the risk of hemorrhagic transformation [9].

Surgical or endovascular interventions are not routinely indicated for an isolated aortic plaque presumed to be responsible for stroke [10]. They are mainly considered when atherosclerosis is associated with aortic obstruction, aneurysmal disease, or another specific anatomical indication [1]. In addition, manipulation of the aorta may expose the patient to a risk of perioperative embolization [10,11]. Therefore, a medical secondary prevention strategy was favored, consisting of atorvastatin 80 mg daily, aspirin 75 mg daily, and strict control of cardiovascular risk factors, with no indication for direct intervention on the aortic plaques.

As the distal arch/isthmic plaque was located downstream from the left subclavian artery, its attribution as a cerebral embolic source would require evidence of retrograde flow reaching a relevant supra-aortic vessel; such a connection was not assessed in our patient [12]. Furthermore, although telemetry and 72-hour Holter monitoring did not detect atrial fibrillation, monitoring beyond 72 hours was not performed, and paroxysmal atrial fibrillation could therefore not be definitively excluded [13].

This case has several limitations. Although representative brain and intracranial vascular images were available, the left V4 stenosis was not quantitatively assessed using a validated intracranial method, and its residual lumen, lesion length, plaque morphology, distal flow, and collateral circulation were not fully characterized. Plaque mobility and definite ulceration were not documented on transesophageal echocardiography. In addition, the exact distance between the distal arch/isthmic plaque and the left subclavian ostium was unavailable, and retrograde aortic flow was not assessed; therefore, retrograde cerebral embolization from this downstream plaque could not be established. The aortic atheroma may consequently have been an incidental finding or a marker of systemic atherosclerosis rather than the direct embolic source. Moreover, in the absence of dedicated coronary imaging and comprehensive peripheral arterial assessment, subclinical atherosclerosis in these territories could not be excluded.

Despite these limitations, this case illustrates the importance of integrating infarct distribution with intracranial vascular anatomy when several potential embolic or atherosclerotic abnormalities coexist. It also shows that complex aortic atheroma may be incidental or may primarily represent a marker of systemic atherosclerosis warranting intensive secondary prevention.

## Conclusions

This case illustrates the difficulty of determining stroke etiology when intracranial left V4 stenosis and complex aortic atheroma coexist. Given the exclusively posterior distribution of the infarcts and the termination of the right vertebral artery as the posterior inferior cerebellar artery, the subocclusive left V4 lesion was considered the more plausible mechanism, potentially through artery-to-artery embolism, distal hypoperfusion, or both. However, the respective contributions of the vertebral and aortic lesions could not be definitively established, and the stroke remained classified as of undetermined etiology under the TOAST framework. The aortic atheroma may have been contributory, incidental, or a marker of systemic atherosclerosis rather than the direct embolic source, particularly because the relevant plaque was distal to the left subclavian artery and retrograde aortic flow was not assessed. This case emphasizes the importance of integrating infarct distribution, vascular anatomy, plaque characteristics, and flow patterns when evaluating competing stroke mechanisms and considering thoracic aortic plaque assessment when the etiology remains uncertain.
